# The *Salmonella* effector protein SpvC, a phosphothreonine lyase is functional in plant cells

**DOI:** 10.3389/fmicb.2014.00548

**Published:** 2014-10-17

**Authors:** Christina Neumann, Malou Fraiture, Casandra Hernàndez-Reyes, Fidele N. Akum, Isabelle Virlogeux-Payant, Ying Chen, Stephanie Pateyron, Jean Colcombet, Karl-Heinz Kogel, Heribert Hirt, Frédéric Brunner, Adam Schikora

**Affiliations:** ^1^Research Center for BioSystems, Land Use and Nutrition, Institute for Phytopathology and Applied Zoology, Justus-Liebig University GiessenGiessen, Germany; ^2^Department of Plant Biochemistry, Center for Plant Molecular Biology (ZMBP), Eberhard Karls University TübingenTübingen, Germany; ^3^Institut National de la Recherche Agronomique, UMR1282 Infectiologie et Santé PubliqueNouzilly, France; ^4^Université François Rabelais de Tours, UMR1282 Infectiologie et Santé PubliqueTours, France; ^5^Unité de Recherche en Génomique Végétale, Plant GenomicsEvry, France

**Keywords:** T3SS, trans-kingdom pathogenicity, *Salmonella*, plant infection

## Abstract

*Salmonella* is one of the most prominent causes of food poisoning and growing evidence indicates that contaminated fruits and vegetables are an increasing concern for human health. Successful infection demands the suppression of the host immune system, which is often achieved via injection of bacterial effector proteins into host cells. In this report we present the function of *Salmonella* effector protein in plant cell, supporting the new concept of trans-kingdom competence of this bacterium. We screened a range of *Salmonella* Typhimurium effector proteins for interference with plant immunity. Among these, the phosphothreonine lyase SpvC attenuated the induction of immunity-related genes when present in plant cells. Using *in vitro* and *in vivo* systems we show that this effector protein interacts with and dephosphorylates activated *Arabidopsis* Mitogen-activated Protein Kinase 6 (MPK6), thereby inhibiting defense signaling. Moreover, the requirement of *Salmonella* SpvC was shown by the decreased proliferation of the Δ*spvC* mutant in *Arabidopsis* plants. These results suggest that some *Salmonella* effector proteins could have a conserved function during proliferation in different hosts. The fact that *Salmonella* and other *Enterobacteriaceae* use plants as hosts strongly suggests that plants represent a much larger reservoir for animal pathogens than so far estimated.

## Introduction

Various pathogenic bacteria such as *Salmonella enterica*, *Pseudomonas aeruginosa*, *Staphylococcus aureus*, *Escherichia coli* O157:H7, and *Listeria monocytogenes* are able to proliferate on both animal and plant organisms (Prithiviraj et al., 2005; Milillo et al., 2008; Schikora et al., 2008, 2011; Haapalainen et al., 2009; Holden et al., 2009). *Salmonella* is a genus of Gram-negative enteropathogenic bacteria that colonizes a wide range of hosts, including humans. These bacteria are the causal agents of gastroenteritis and typhoid fever (Pang et al., 1995). The most common mode of infection in humans is the ingestion of contaminated food or water. Whereas 0.3% of fresh products were contaminated with *Salmonella* bacteria in 2007 in the European Union (Westrell et al., 2009), the proportion of raw-food related outbreaks reached 25% in the USA in recent years (Rangel et al., 2005).

The study of the *Salmonella* infection mechanism was until recently mainly driven by its medical aspect; therefore the mouse and human epithelial cell models are the best studied to date. Today, it is still poorly understood how these bacteria successfully proliferate in such diversified hosts as animals or plants. However, important insights were obtained during last years. Stomata openings were identified as possible entry points of bacteria into the inner layers of the mesophyll (Kroupitski et al., 2009). Interestingly, while some plant species (e.g., arugula) allow the *Salmonella enterica* subsp. *enterica* ser. Typhimurium (*S*. Typhimurium) strain SL1344 to internalize, some others (e.g., parsley) seem to be capable of preventing internalization (Golberg et al., 2011). In a previous report, we showed that in *Arabidopsis thaliana*, roots and especially root hair cells can be colonized by *Salmonella* (Schikora et al., 2008).

Studies of the infection mechanisms in animals revealed that, besides remodeling the host cell architecture, *Salmonella* actively suppresses the host immune system by injecting a cocktail of effector proteins. These effectors are delivered by Type III Secretion Systems (T3SSs). *S*. Typhimurium possesses two distinct T3SSs, T3SS-1, and T3SS-2, encoded by two *Salmonella* Pathogenicity Islands, SPI-1 and SPI-2, respectively. To date, about 44 *Salmonella* effectors have been described and the function of many of them is known [reviewed in Heffron et al. (2011)]. In addition to SPIs, some *Salmonella* serovars carry plasmids with a common locus called salmonella plasmid virulence (*spv*) (Boyd and Hartl, 1998). The *spv* operon encodes further effector proteins responsible for full virulence in humans and in the mouse model (Montenegro et al., 1991; Fierer et al., 1992; Gulig and Doyle, 1993; Chu and Chiu, 2006).

Even though some *Salmonella* effectors have homologs in plant pathogenic bacteria, the role of *Salmonella* T3SS-dependent effectors in the modulation of the plant immune system and their contribution to plant host susceptibility are less understood. Plants induce defense mechanisms after recognition of pathogens. This recognition may occur at two levels: (i) at the cell surface, where Pattern Recognition Receptors (PRRs) recognize conserved microbial structures called Pathogen-Associated Molecular Patterns (PAMPs), and (ii) in the cytoplasm where Resistance (R) proteins recognize bacterial effectors injected into plant cells. Both recognition events initiate immune responses referred to as Pattern-Triggered Immunity (PTI) [renamed from PAMP-triggered immunity (Boller, 2012)] or Effector-Triggered Immunity (ETI), respectively. An activation of MAPKs and enhanced expression of *Pathogenesis Related* (*PR*) genes are hallmarks of both: the PTI and the ETI responses. Both responses were already observed after inoculation with *Salmonella* (Schikora et al., 2008; Meng et al., 2013; Garcia et al., 2014). Recently, the suppression of plant defense by *Salmonella* was reported in two different systems. In contrast to living *S.* Typhimurium, treatment with dead or chloramphenicol-treated bacterial cells elicited an oxidative burst and changes in apoplastic pH in tobacco (Shirron and Yaron, 2011). Similar responses were provoked by inoculation with the *invA* mutant, which has no functional T3SS-1, showing that T3SS-deficient or dead bacteria induce defense reactions while living wild-type bacteria actively suppress their induction. We observed a very similar phenomenon in *Arabidopsis* plants (Schikora et al., 2011). Inoculation with wild-type *S.* Typhimurium strain 14028s provoked changes in expression of 249 and 1318 genes at 2 and 24 h after infection, respectively (Schikora et al., 2011). However, inoculation with the *prgH* mutant, which has no functional T3SS-1, changed the expression of over 1600 genes at 24 h. Gene ontology (GO) term enrichment analysis of the 649 *prgH*-specific genes revealed an overrepresentation of genes related to pathogen responses and ubiquitin-mediated protein degradation (Schikora et al., 2011; Garcia et al., 2014). Interestingly, this set includes *BAK1*, *BIK1*, *WRKY18*, *WRKY33*, *EIN3*, *PR4*, *FRK1*, *4CL*, *Sec61*, and *PUB23*, all of which are up-regulated upon inoculation with pathogen or PAMP treatment. The higher expression levels of these genes after inoculation with the *prgH* mutant compared to the wild-type imply that the mutant is lacking an effective suppression mechanism to hinder plant defense. A powerful response to pathogen attack is the hypersensitive response (HR). This induced cell death is often the reaction to bacterial proteins present in the host cytoplasm (Jones and Dangl, 2006). In respect to *Salmonella* effector proteins, SseF was the first effector reported to induce HR-like symptoms in tobacco plants (Ustun et al., 2012). The fact that SseF-induced HR-like symptoms can be suppressed by RNAi-mediated silencing of *SGT1* (*Suppressor of G2 allele of Skip1*) indicates an R-protein-mediated response, identical to ETI.

In this report, we present two functional screens of *Salmonella* effector proteins and virulence factors in plants. Our screens resulted in the identification of the phosphothreonine lyase SpvC, which was able to suppress PTI. Using *in vitro* and *in vivo* systems we showed that this effector protein actively interacts with and dephosphorylates activated *Arabidopsis* Mitogen-activated Protein Kinase 6 (MPK6). MAPKs are important regulators of the immune response in animals and plants and the dephosphorylation of MPK6 hinders the induction of defense-related genes in *Arabidopsis*. Moreover, we showed that bacterial fitness on *Arabidopsis* plants is compromised in mutants lacking the *SpvC* gene. These results strengthen the notion that some *Salmonella* effectors may be equally applied in plant and animal systems to suppress the respective host immune systems.

## Materials and methods

### Plant growth conditions

*Arabidopsis thaliana* Colombia-0 (N60000) plants were cultivated on soil under stable climate conditions: 8 h light/16 h dark at 20°C, 40–60% humidity, ~120 μE m-2 s-1 light intensity. Leaves from 4-week old plants were used for protoplast preparation and analysis of transient gene expression. Alternatively *Arabidopsis* seedlings were germinated on sterile half-strength MS agar medium and cultivated for 2 weeks in short-day conditions (at 21°C, 60% humidity) in growing chambers. *Nicotiana benthamiana* plants were germinated and cultivated on soil, in a greenhouse under long-day conditions (16 h light at 22°C, 40–60% humidity) for 4 weeks.

### Cloning of *Salmonella* virulence factors and SPI-dependent effector proteins

Fifty-four *Salmonella* virulence genes, which when mutated caused the attenuation of virulence in the mouse model, and genes coding 18 SPI-1- or SPI-2-encoded effectors from *Salmonella enterica* subsp. *enterica* serovar Typhimurium strain 14028s (*S*. Typhimurium) were cloned into the versatile Gateway (Invitrogen) vector system. All open reading frames (ORFs) were constructed in two versions: one including the native stop codon: the STOP version and a second without the stop codon: the END version. Cloning was based on the ATOME cloning strategy (http://urgv.evry.inra.fr/ATOMEdb). The consequential entry clones were sequenced and those with correct ORFs were used for further studies. For the screen in *Arabidopsis* protoplasts, the ORFs were further recombined into p2GW7 (VIB, University of Ghent). *SpvC* was additionally cloned into p2FGW7 (VIB, University of Ghent) for expression of the N-terminal GFP fusion protein GFP-SpvC.

### Bacterial mutagenesis

The *SpvC* mutant Δ*spvC* of the *S*. Typhimurium 14028 strain was obtained using the λ-Red mutagenesis system as described by Datsenko and Wanner (2000). The sequences of the primers used were: 5′ATGCCCATAAATAGGCCTAATCTAAATCTAAACATCCCTCCTTTGAATATGTGTAGGCTGGAGCTGCTTC3′ and 5′TTACTCTGTCATCAAACGATAAAACGGTTCCTCACGTAAAGCCTGTCTCTCATATGAATATCCTCCTTAG3′.

### *Agrobacterium*-mediated transformation

The Gateway compatible pGreen derivative vectors pJC005 and pJC001 for expression of 10xMyc- or 3xHA-tagged recombinant proteins, respectively, carrying *Salmonella* ORFs, were transformed into the *Agrobacterium tumefaciens* strain GV3101, pMP90. Transformed bacteria were cultivated until stationary phase, washed in infiltration medium (10 mM MgCl_2_, 10 mM MES-KOH, pH 5, 4, 200 μM acetosyringone) and incubated for 2 h in the dark. OD_600_ of the infiltration solution was then adjusted to 0.3. Leaves of *N. benthamiana* were infiltrated one-sided.

### Protoplast transformation

The preparation of *Arabidopsis* mesophyll protoplasts was performed according to the protocol from Yoo et al. (2007) with minor changes (Fraiture et al., 2014). Briefly, thin leaf stripes were dipped into 1.5% cellulose “Onozuka” R10—0.4% macerozyme R10 solution (Yakult Pharmaceutical Industry), vacuum-infiltrated for 30 min and digested for 3 h at 20°C in the dark. After two subsequent washing steps with W5 buffer *Arabidopsis* protoplasts were suspended to a concentration of 2 × 10^5^ cells/ml in MMG buffer and subjected to polyethylene glycol-mediated transfection. 100 μg plasmid DNA/ml protoplast suspension was used during transfection. Protoplasts samples were then incubated in W1 buffer at 20°C in the dark for 12–16 h allowing plasmid gene expression.

### Luciferase reporter gene assays

Luciferase gene assays were conducted to screen for immunity-suppressing effects of effector proteins from *Salmonella* (Fraiture et al., 2014). For this, *Arabidopsis* protoplasts were co-transfected with *pFRK1-Luciferase* (*pFRK1-Luc*) and a candidate effector gene in p2GW7 (or empty p2FGW7 serving as GFP control). For the assay, luciferin was added to 600 μl transfected protoplast solution to a final concentration of 200 μM. Protoplasts were transferred to an opaque 96-well plate (100 μl per well). For each sample, flg22 was added to 3 wells to a final concentration of 500 nM. The remaining 3 replicates were left untreated. The luminescence reflecting the luciferase activity was measured at different time-points using a Berthold Mithras LB 940 luminometer.

### RNA isolation and quantitative real-time PCR

Total RNA from 400 μl protoplast solution was extracted with TRI reagent (Ambion) and treated with DNase I (Macherey-Nagel) following the suppliers' protocols. Poly A-tailed RNA (1 μg) was converted to cDNA using the RevertAid reverse transcriptase (Fermentas) and oligo-dT primers. qRT-PCR reactions were performed in triplicates with the Maxtra SYBR Green Master Mix (Fermentas) and run on a Biorad iCycler according to the manufacturer's instructions. The primers used for the qRT-PCR are presented in Supplementary Table S1. Relative gene expression was determined with a serial cDNA dilution standard curve. The actin transcript was used as an internal control in all experiments. Data was processed with the iQ software (Biorad) (Zheng et al., 2014).

### Immunoblot analysis

To monitor the activation of MAPKs, *Salmonella* effector-gene transformed protoplasts were challenged with 500 nM flg22 (Zheng et al., 2014). Pellets from 100 μl protoplast solution were collected 0, 15, and 30 min after treatment and dissolved in denaturating protein loading buffer. Proteins were separated by SDS-PAGE, blotted onto nitrocellulose membranes (Hybond–ECL, Amersham) and stained with 0.1% Ponceau S to visualize equal sample loading. The membranes were incubated with anti-phospho-p44/42 MAPK antibody (Cell Signaling Technology) diluted 1/1000 in 5% BSA TBS-T. The expression of GFP-tagged *Salmonella* virulence proteins and effectors was assessed in *Arabidopsis* protoplasts collected 24 h after transformation using an anti-GFP antibody. The immunoblot was revealed in NBT/BCIP detection solution.

### Protein purification

Recombinant GST-SpvC and 6xHis-SpvC proteins were produced in *E. coli* BL21 bacteria using the pDEST15 and pDEST17 vectors (Invitrogen). Protein expression was induced with 1 mM IPTG overnight at 30°C. Cells were lysed and protein purified accordingly to the manufacturers' protocols (Macherey-Nagel for GTH-beads and Qiagen for Ni-beads purifications).

### Pull-down assay

For the pull-down assay 50 μg of purified recombinant proteins were incubated with 50 μg of total *Arabidopsis* protein extract in the presence (or absence) of 80 μg BSA for 30 min in a final volume of 200 μl at 21°C together with the corresponding beads. Beads were washed 3 times and Ni- or GTH- binding complexes separated by SDS-PAGE. Anti-MPK6, anti-MPK3, or anti-MPK4 antibodies (Sigma-Aldrich) were used to visualize the binding between SpvC and MAPKs.

### BiFC

Bimolecular fluorescence complementation (BiFC) assay was performed using the full-length versions of *MAPKs* and *SpvC* cloned down-stream of N-terminal or C-terminal part of gene coding for the *Yellow Fluorescent Protein* (*YFP*) in both combinations, using pBIFC1-4 vectors. *Arabidopsis* epidermal cells were co-transformed with vectors carrying those constructs and vector carrying *p35S-mCherry*. Fluorescence was observed 24–48 h after transformation. Expression of *mCherry* was used as readout for successful transformation. Reconstitution of functional YFP was observed with the 510–540 nm band pass filter on a Leica SP2 confocal laser-scanning microscope.

### *In vitro* dephosphorylation assay

The phosphatase activity of SpvC on activated MAPKs was assessed using 25 μg purified recombinant GST-SpvC or 6xHis-SpvC proteins and 50 μg of total protein extract from *Arabidopsis* seedlings treated or not (control) with 1 μM flg22 for 15 min. Recombinant effector proteins and *Arabidopsis* proteins were co-incubated for 30 min at 21°C. Samples were precipitated using a chloroform/methanol procedure and separated by SDS-PAGE. The presence of the phosphorylated pTEpY epitope was probed with anti-pERK1/2 antibody (see above).

### Pathogenicity assay

To assess the *Salmonella* proliferation rate in plants, soil-grown, 4-week old *Arabidopsis* Col-0 plants were infiltrated with wild-type *Salmonella enterica* subsp. *enterica* ser. Typhimurium strain 14028s or its isogenic mutant Δ*spvC*, using syringe infiltration. Bacteria were grown in LB medium until early log phase, washed and re-suspended in 10 mM MgCl_2_. Infiltration solution was adjusted to OD_600_ = 0.01 (1.7 × 10^6^ bacteria/ml). Bacterial population was monitored during 4 days post-infiltration as described in Schikora et al. (2008).

### Incompatible interaction

To test the breach of non-host resistance, leaves from soil-grown *Arabidopsis* plants were transformed with *p35S-GFP-SpvC* or *mCherry* via particle bombardment and inoculated with *Blumeria graminis f. sp. hordei* (*Bgh*) conidia. After 48 h, leaves were stained with calcofluor to visualize fungal growth. The outcome of interaction was counted on cells transformed either with *mCherry* (control) or plasmid carrying *GFP-SpvC*.

## Results

### A dual screen for *Salmonella* virulence factors and effector proteins active in plant cells

**Figure 1:**
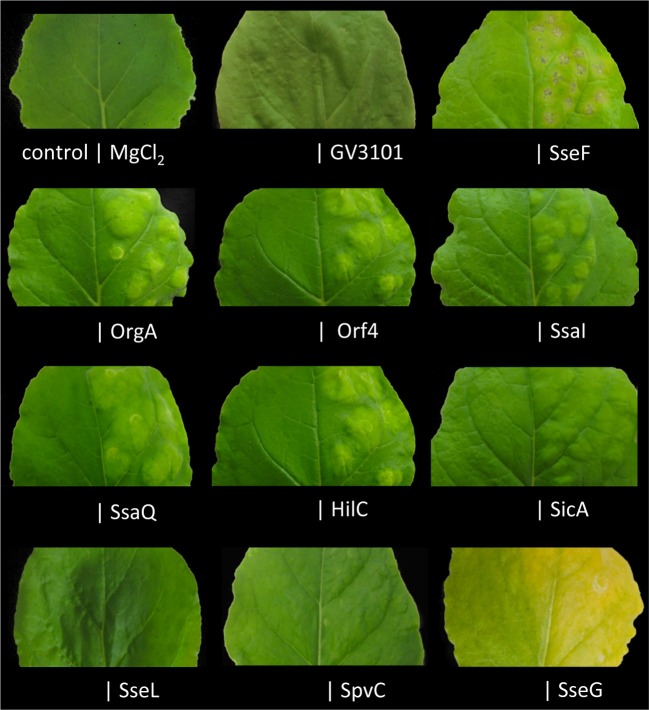
**Screen for *Salmonella* proteins that induce HR-like symptoms in plants**. SVF and effector genes were cloned into plant expression vectors and expressed as HA- and Myc-tagged versions in *N. benthamiana* leaves *via Agrobacterium*-mediated transformation. Symptoms were observed 5 days after infiltration. Expression of eight proteins resulted in macroscopic changes in leaf morphology when compared to the non-transformed parts of the leaf (control), transformation with *Agrobacterium* GV3101 or infiltration with 10 mM MgCl_2_. The experiment was repeated 4 times with both versions of bacterial fusion protein. Only the right side of the leaf was infiltrated.

To identify the important factors for *Salmonella* pathogenicity on plants we decided to follow a two-screening-strategy through a set of *Salmonella* virulence factors (SVFs) and *Salmonella* Pathogenicity Islands (SPIs)-encoded effector proteins. We chose 54 SVF genes, which when mutated caused an attenuation of virulence in the mouse model (PHI-base, www.phi-base.org), and 18 SPI-1- or SPI-2-encoded effectors (Heffron et al., 2011) from *Salmonella enterica* subsp. *enterica* ser. Typhimurium strain 14028s (*S*. Typhimurium) for cloning into the versatile Gateway (Invitrogen) vector system. Cloned genes resulted in a set of *Salmonella* ORFs used for further studies. In a first step, 37 ORFs were successfully cloned into binary vectors for *Agrobacterium*-mediated expression in tobacco (*Nicotiana benthamiana*) leaves. *Salmonella* SVFs and effectors were expressed as N-terminal 10xMyc or 3xHA fusion proteins and symptoms caused by the expression were observed during 5 days after infiltration. We identified eight proteins (SseF, OrgA, Orf4, SsaI, SsaQ, HilC, SicA, and SseG), which caused chlorosis, wilting or hypertrophy on tobacco leaves (Figure 1), while the expression of the others, provoked no visible symptoms (Table 1).

**Table 1:** **Salmonella ORFs cloned for screens in plants**.

**Gene**	**Tobacco assay**		**Protoplast assay**
	**10xMyc**	**3xHA**	
***Salmonella* VIRULENCE FACTORS**			
*ssrB*	–	–	
*ssaB*	–	–	
*sseE*	–	–	
*sseF*	HR	HR	PTI suppression
*sifA*	–	–	
*sirA*	–	–	
*orgA*	Hypertrophy	Hypertrophy	–
*orf2*	–	–	
*ttrB*	–	–	
*ssaM*	–	–	
*orf2*	–	–	
*orf4*	Hypertrophy	Hypertrophy	–
*ssaE*	–	–	
*sseD*	–	–	
*sscB*	–	–	
*ssaI*	Hypertrophy	Hypertrophy	–
*ssaJ*	–	–	
*ssaK*	–	–	
*ssaM*	–	–	
*ssaQ*	Hypertrophy	Hypertrophy	–
*yscR*	–	–	
*ssaS*	–	–	
*ssaT*	–	–	
*hilC*	Hypertrophy	Hypertrophy	–
*prgK*	–	–	
*prgJ*	–	–	
*iagB*	–	–	
*sicA*	Hypertrophy	Hypertrophy	Weak PTI suppression
*invI*	–	–	
*invE*	–	–	
*spvR*	–	–	
***Salmonella* T3SS-1 AND T3SS-2 DEPENDENT EFFECTORS**			
*avrA*	–	–	–
*sptP*	–	–	–
*slrP*	–	–	–
*sseL*	–	–	PTI suppression
*spvC*	–	–	PTI suppression
*sseG*	Yellowing	Yellowing	PTI suppression

Symptoms observed on tobacco leaves 5 days after infiltration with Agrobacterium tumefaciens (tobacco assay) or suppression of the pFRK1-Luc activity in protoplasts challenged with flg22 (protoplast assay). HR, hypersensitive response; –, represents no difference to controls.

**Figure 2:**
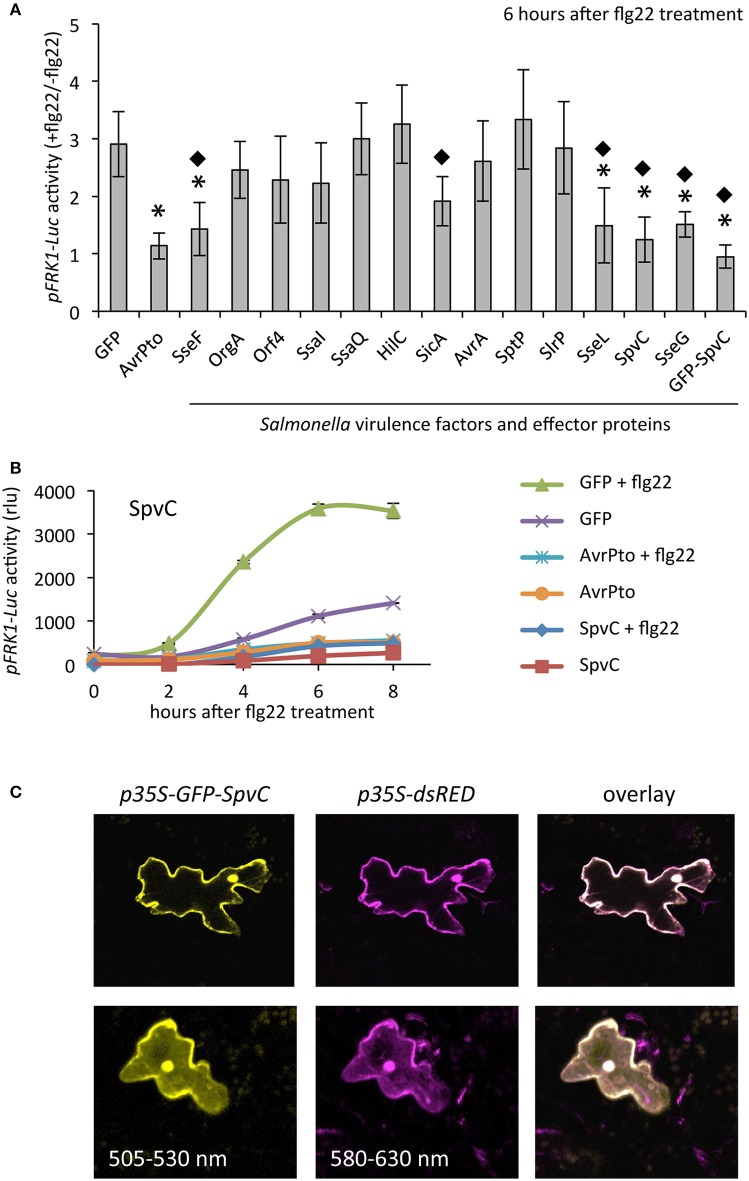
**Suppression of flg22-induced *pFRK1-Luc* expression by *Salmonella* proteins. (A)** Mesophyll protoplasts from *Arabidopsis thaliana* Col-0 were co-transformed with pFRK1-Luc and p35S-Salmonella-ORF plasmids. Co-transformations of pFRK1-Luc with p2FGW7 (GFP) and with p2GW7-AvrPto (AvrPto) plasmids served as controls. Protoplasts were subsequently treated with flg22 or left untreated. The ability to suppress the flg22-driven activation of *pFRK1-Luc* of chosen virulence factors and effectors was assessed 6 h later by measuring luciferase (Luc) activity. Results are presented as ratio between flg22-treated and non-treated samples (+flg22/−flg22). For each effector, at least four independent experiments with three technical replicates were carried out. All data were pooled. Mean values ± SD are plotted. One-way ANOVA followed by Dunnett's multiple comparison test was performed to assess significant differences between the GFP control and the virulence factor- and effector- protein-producing samples. An asterisk marks data sets with *p* < 0.01. The same test was performed to assess the difference between *Salmonella* effectors and AvrPto. A diamond represents those proteins, which have similar effects to AvrPto at *p* > 0.05. **(B)** Representative time-course experiment of flg22-mediated *pFRK1-Luc* activity in *Arabidopsis* protoplasts expressing *GFP*, *AvrPto* or *SpvC*. Luciferase activity was measured every 2 h for 8 h after flg22 challenge. The data represents mean values ± SD from three technical replicates. rlu; relative light units. **(C)** Localization study of a GFP-SpvC fusion protein produced under the 35S promoter in *Arabidopsis* leaves transformed via particle bombardment. Cytoplasmic and nuclear localized dsRED protein was used as a control. Images present two exemplary cells (lower and upper panels, respectively) expressing the GFP-SpvC fusion protein.

Next, we tested the potential of the proteins inducing visible changes in tobacco leaves for suppressing early defense responses. Additionally, we tested 5 selected *Salmonella* effectors (AvrA, SptP, SlrP, SseL, SpvC) for which the biochemical function and/or suppression of immunity in mammals has well been characterized (Heffron et al., 2011). We used a protoplast-based system in *Arabidopsis* in which transiently expressed effectors were evaluated for their capability to suppress PAMP-triggered activation of luciferase (Luc) activity. In our screen *Luc* expression was driven by the *FRK1* promoter, which is strongly induced upon treatment with the PAMP flg22, a 22 amino acid long peptide derived from the N-terminal part of flagellin and conserved in many pathogenic bacteria including *Pseudomonas aeruginosa*, *Escherichia coli* and *S*. Typhimurium (Felix et al., 1999). Out of the 13 tested *Salmonella* proteins, a strong suppression of *pFRK1-Luc* activity 6 h after flg22 treatment was observed when co-expressing the SpvC effector protein (*p* < 0.01, one-way ANOVA and Dunnett's test) compared to the GFP control (Figure 2A and Supplementary Figure S1). The suppression effect was comparable (*p* > 0.05) with AvrPto from *Pseudomonas syringe*, an effector that is known to interfere with early PAMP signaling (He et al., 2006). In addition, significant suppression (*p* < 0.01) was observed when expressing *SseL*, *SseG*, and *SseF* (Figure 2A). In order to confirm our observations we performed a time-course experiment, in which we analyzed *pFRK1-Luc* activity in *SpvC*-transformed protoplasts during 8 h after induction with flg22 (Figure 2B). SpvC and AvrPto have similar effects on the activity of *pFRK1* suggesting that SpvC may, similarly to AvrPto, affect PAMP signaling at an early stage (4 h or earlier). Comparable observations were made when the fusion protein Green Fluorescent Protein-SpvC (GFP-SpvC) protein was expressed (Figure 2A). The localization analysis performed with GFP-SpvC fusion protein indicated that the effector protein localizes to the cytoplasm and nucleus when present in plant cells (Figure 2C). In the following experiments we decided to focus on SpvC, because of its well-known inhibitory effect on immunity during animal infection (Mazurkiewicz et al., 2008).

### *Salmonella* SpvC effector suppresses the expression of PAMP-induced genes

**Figure 3:**
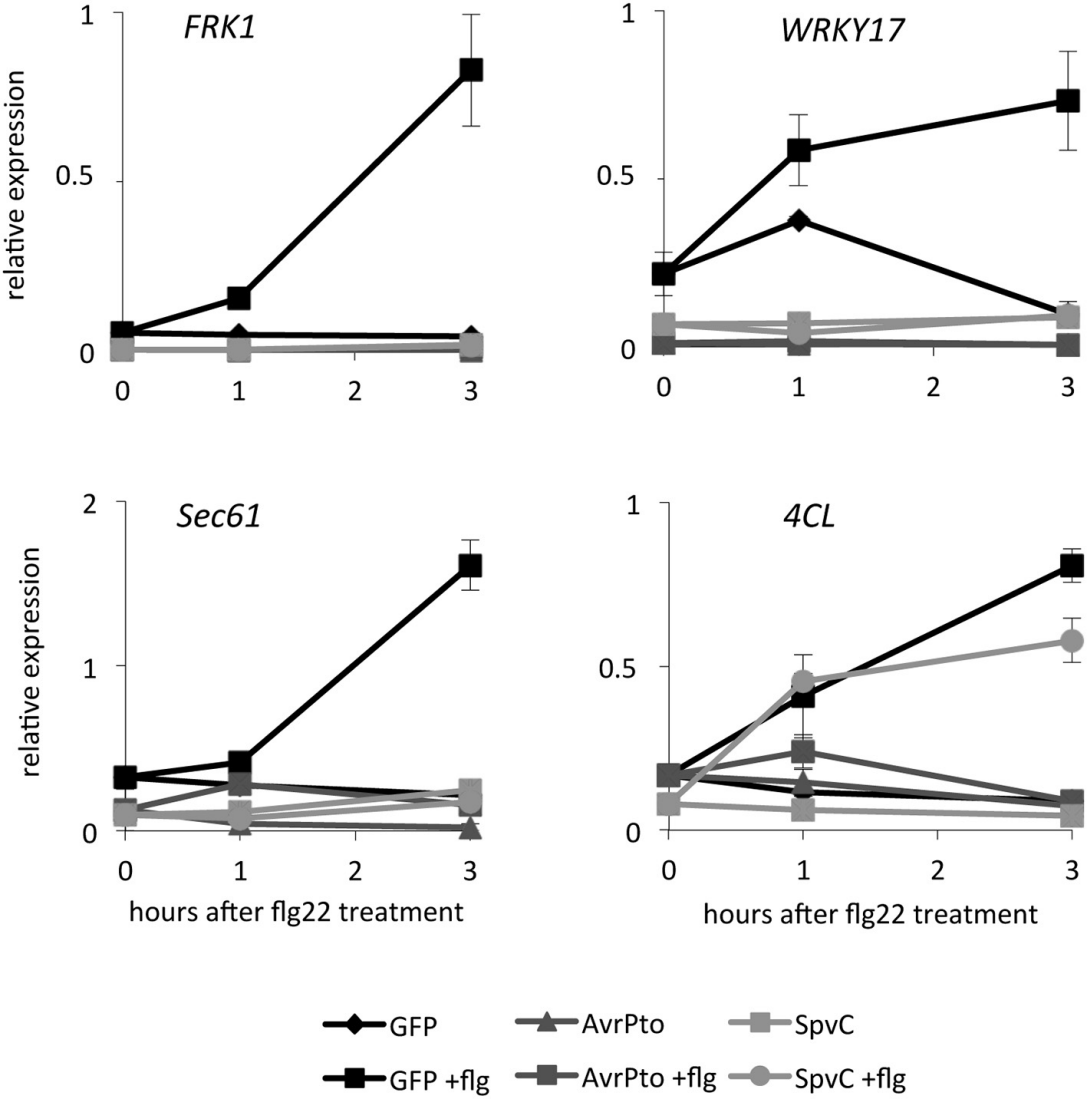
**SpvC attenuates flg22-induced defense responses in protoplasts**. *Arabidopsis* mesophyll protoplasts were transformed with p2FGW7 (GFP), p2GW7-AvrPto (AvrPto) or p2GW7-SpvC (SpvC) plasmids and subsequently challenged with flg22. Samples were collected 1 and 3 h after treatment. Relative expression levels of *FRK1*, *WRKY17*, *Sec61*, and *4CL* were assessed using quantitative RT-PCR and normalized to the expression of the house-keeping gene *actin*. The graphs show one representative experiment out of three. Data is presented as mean values ± s.e.m. of three technical replicates. Expression of *FRK1*, *WRKY17*, and *Sec61* was attenuated in protoplasts in the presence of SpvC. However, SpvC had no impact on the expression of *4CL*.

In a next step, we analyzed the effect of SpvC on the activity of the endogenous *FRK1* promoter. To this end, we measured the expression of *FRK1* in *Arabidopsis* protoplasts transformed with *SpvC*, *AvrPto*, or *GFP* after 1 and 3 h challenge with flg22. Equally to previous experiments and in accordance with the literature (Asai et al., 2002), expression of *FRK1* was induced upon treatment with flg22. Expression of *AvrPto* efficiently suppressed this induction in *Arabidopsis* protoplasts (He et al., 2006). Likewise, SpvC also abolished the induction of endogenous *FRK1* expression after flg22 treatment (Figure 3). We extended our analysis to other PAMP-induced genes. Similar to *FRK1*, the transcription factor *WRKY17* and the gene encoding for the protein transport protein Sec61 were induced upon flg22 treatment and hindered in their inductions by AvrPto and SpvC (Figure 3). Remarkably, SpvC did not suppress all tested PAMP-induced genes. The *4CL* gene encoding a 4-coumarate-CoA ligase was induced after flg22 treatment and repressed in the presence of AvrPto. However, in contrast to AvrPto, SpvC was not able to restrain its flg22-driven induction (Figure 3). These results suggest that the *Salmonella* effector SpvC interferes only with a subset of flg22-induced defense related genes (*FRK1*, *WRKY17*, and *Sec61*, but not *4CL*).

### Interaction between *Arabidopsis* MPK6 and *Salmonella* SpvC

**Figure 4:**
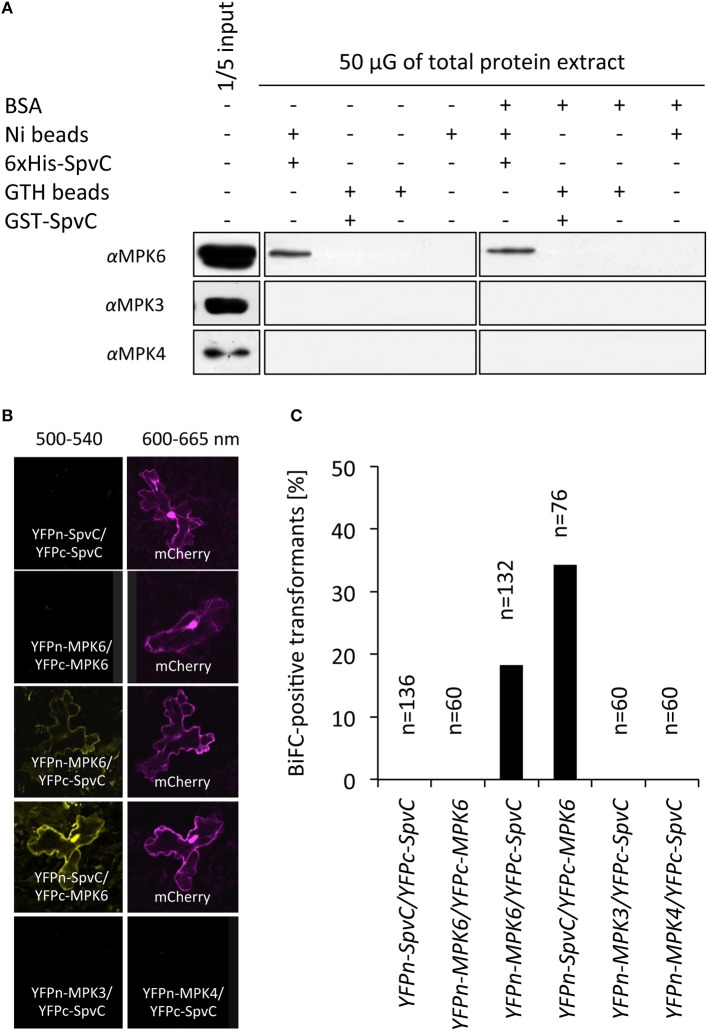
**Arabidopsis MPK6 interacts with the SpvC effector. (A)** In a pull-down assay, recombinant 6xHis-SpvC or GST-SpvC proteins were co-incubated with total protein extract from *Arabidopsis* seedlings and Ni- or GTH-coated beads, respectively, as indicated by “+”. Anti-MPK6, anti-MPK3, and anti-MPK4-specific antibodies were used to visualize the presence of the respective kinases in Ni- or GTH-binding complexes in an immunoblot analysis. Only MPK6 was detected, indicating a specific binding between MPK6 and 6xHis-SpvC, however not GST-SpvC, proteins. **(B)** Bimolecular fluorescence complementation (BiFC) assay was performed with full-length versions of *MAPKs* and *SpvC* cloned down-stream of a portion of the *Yellow Fluorescent Protein* (*YFP*) gene encoding the N-terminal or C-terminal part of YFP in all four possible combinations. *Arabidopsis* epidermal cells were co-transformed with vectors carrying those constructs and vector carrying *35S-mCherry* via particle bombardment. Fluorescence was observed 48 h after transformation. **(C)** Quantification of the interaction between SpvC and MAPKs as percentage of the transformed cells. mCherry-positive cells from four independent experiments were counted. The diagram represents the percentage of YFP-positive cells among transformed cells.

Inhibition of flg22-induced gene expression by bacterial effectors can occur at many levels from the flg22 receptor complex (FLS2-BAK1), through the MAPK cascade, down to transcriptional regulation of defense genes. MAPK cascades play a key role in flg22 signal transduction and in pathogen defense. Among the 20 *Arabidopsis* MAPKs, MPK3, MPK4, and MPK6 are strongly activated by flg22 (Asai et al., 2002; Pitzschke et al., 2009). Based on the functional characteristics of SpvC during animal infection as well as the function of other members of the OspF family [e.g., HopAI1 (Zhang et al., 2007)], we hypothesized that SpvC targets plant MAPKs. To test our hypothesis, we analyzed possible protein-protein interactions between SpvC and *Arabidopsis* MAPKs. Recombinant 6xHis-SpvC and GST-SpvC proteins were expressed and purified from *E. coli* BL21 cells. The recombinant proteins were subsequently co-incubated with total protein extract from *Arabidopsis* seedlings and either Ni- or GTH-coated beads were used to precipitate the respective Ni- or GTH-binding complexes. Pull-down samples were probed for the presence of MAPKs in immunoblot assays. In the presence of His-tagged, but not GST-tagged SpvC, we detected the MPK6 in the pulled-down protein complex (Figure 4A), suggesting the interaction between SpvC and MPK6. This interaction was observed even in the presence of an excess of BSA. However, we did not detect MPK3 or MPK4, indicating a specific interaction between SpvC and MPK6.

The *in vitro* SpvC-MPK6 interaction was tested also in bimolecular fluorescent complementation (BiFC) assays. Full-length cDNAs of *SpvC* and the three *MAPKs* were cloned downstream of sequences encoding either the N- or C-terminal part of the Yellow Fluorescent Protein (YFP) and subsequently transiently expressed in *Arabidopsis* epidermal cells via particle bombardment. Both tested combinations: (i) *YFPn-MPK6* with *YFPc-SpvC* and (ii) *YFPn-SpvC* with *YFPc-MPK6*, when expressed together, resulted in reconstitution of a functional YFP protein (Figure 4B). We co-expressed the constructs with *p35S-mCherry* plasmid, allowing normalization of the interaction events (Figure 4C). Eighteen percent of all transformed cells showed visible interaction between SpvC and MPK6 when *YFPn-MPK6* was co-expressed with *YFPc-SpvC*, and 34% of all cells when *YFPn-SpvC*, and *YFPc-MPK6* were used as interaction partners. *Arabidopsis* MPK6 localizes to the cytoplasm and nucleus, but accumulates in the nuclear compartment after activation (Bethke et al., 2009). The observed cytoplasmic and nuclear localization of SpvC-MPK6 complex had similar localization. Moreover, this localization overlapped with the localization of the GFP-tagged versions of SpvC in epidermal cells (Figure 2C). Similarly to the *in vitro* assay, we did not observe an interaction between SpvC and the other MAPKs (MPK3 and MPK4) (Figure 4B). Hence, these results indicate that SpvC interacts with *Arabidopsis* MPK6.

### Activated MAPKs are dephosphorylated by SpvC

**Figure 5:**
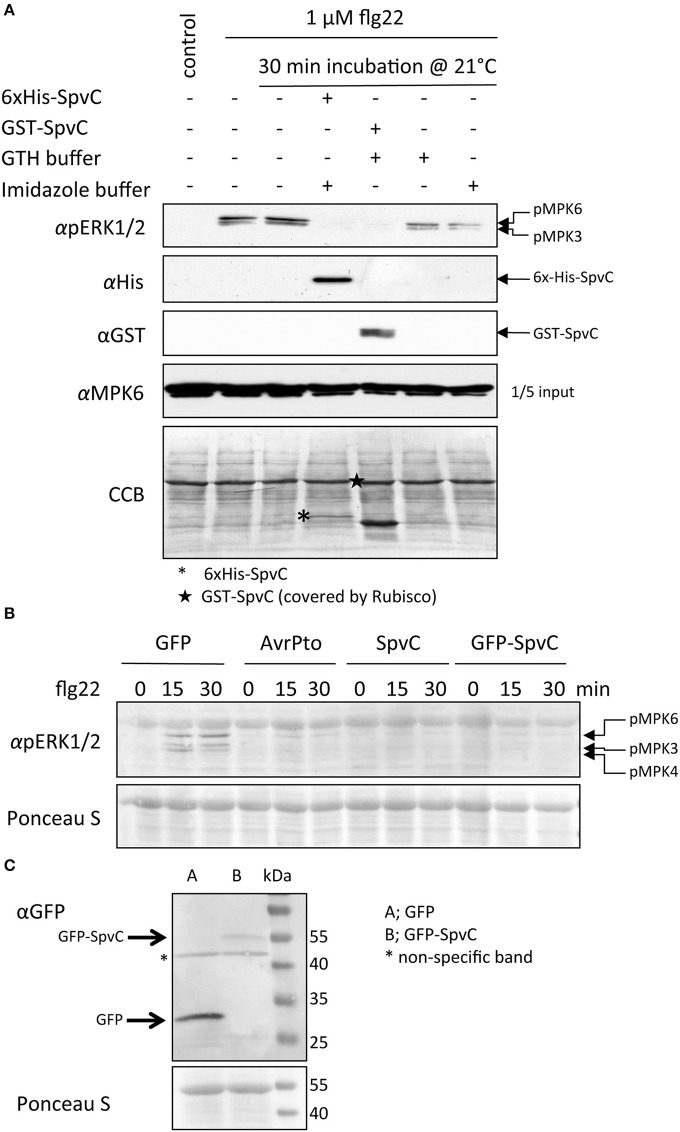
**Active MAPKs are dephosphorylated by SpvC. (A)** MAPKs were activated by treatment of 2-week old seedlings with flg22 for 20 min prior to the extraction of total soluble proteins. Extracted proteins were then incubated for 30 min with purified recombinant 6xHis-SpvC or GST-SpvC proteins. The phosphorylation status of *Arabidopsis* MAPKs was analyzed by immunoblotting using an antibody raised against the phosphorylated form of EKR1/2 (αpERK1/2). The recombinant proteins were probed with anti-His and anti-GST antibodies (αHis and αGST, respectively). Specific anti-MPK6 (αMPK6) antibody was used to assess the presence of MPK6. CCB stain was used to monitor the equal sample loading. **(B)** *Arabidopsis* protoplasts were transformed with p2FGW7 (GFP), p2GW7-AvrPto (AvrPto) or p2GW7-SpvC (SpvC) and subsequently treated with flg22 for 0, 15 or 30 min. The phosphorylation status of MAPKs was assessed with the αpERK1/2 antibody. Treatment with flg22 caused phosphorylation of MPK6 and MPK3 as visible by the appearing bands at 15 and 30 min after treatment. However, signals are missing in protoplasts expressing *AvrPto* or *SpvC*. **(C)** Expression and stability of GFP-SpvC fusion protein in *Arabidopsis* protoplasts. Ponceau S staining was used to show equal sample loading.

By monitoring *in vitro* the phosphorylation status of MAPKs after activation with flg22 in the presence of SpvC, we tested the assumption that SpvC might dephosphorylate the double phosphorylated active forms of MAPKs. The recombinant proteins 6xHis-SpvC and GST-SpvC were expressed in *E. coli* BL21 cells and purified with the respective affinity chromatography (Ni-sepharose or GTH-agarose columns, respectively). *Arabidopsis* MAPKs were activated by challenge of intact seedlings with flg22. Total proteins from those seedlings were incubated with recombinant SpvC protein (Figure 5A). The activation of the MAP kinases can be efficiently detected by means of an anti-pERK1/2 antibody that recognizes the phosphorylated T and Y residues in the activation loop (pTEpY) of MAPKs (Hamel et al., 2005). In Figure 5, the upper panel presents the phosphorylation status of MPK6 (upper band) and MPK3 (lower band) as detected by means of the anti-pERK1/2 antibody (αpERK1/2). Twenty minutes after treatment of *Arabidopsis* seedlings with flg22, the detected signals indicated active, phosphorylated MAPKs. It should be noted that the 30 min incubation time, which is necessary to carry out this assay, did not affect the phosphorylation on the TEY epitope. When 6xHis-SpvC or GST-SpvC proteins were added to the *Arabidopsis* protein extract, the phosphorylated pTEpY epitope of the MAPK was no longer detectable (Figure 5A, αpERK1/2 blot). This result suggests that SpvC is able to dephosphorylate activated plant MAPKs *in vitro*.

In the next step we sought to verify the result in an *in vivo* system. To achieve this aim, we expressed *SpvC* as native or GFP-tagged protein in *Arabidopsis* protoplasts, and subsequently assessed the phosphorylation status of MAPKs after flg22 treatment. As controls we transformed the protoplasts with *GFP* or the effector *AvrPto*, which is known to inhibit MPK6 phosphorylation (He et al., 2006). In *GFP*-expressing protoplasts, flg22-triggered transient activation of MAPKs was peaking after 15 min (Figure 5B). In contrast, protoplasts expressing *AvrPto*, *SpvC* as well as *GFP-SpvC* showed complete inhibition of MAPK activity (Figures 5B,C). These results are in line with the dephosphorylation activity of SpvC observed *in vitro*, as well as the suppressing effect on defense gene activation. Moreover, they support the idea that SpvC interferes with plant defense signaling upstream or at the level of the MAPKs.

### Expression of SpvC breaches the non-host resistance in *Arabidopsis*

**Figure 6:**
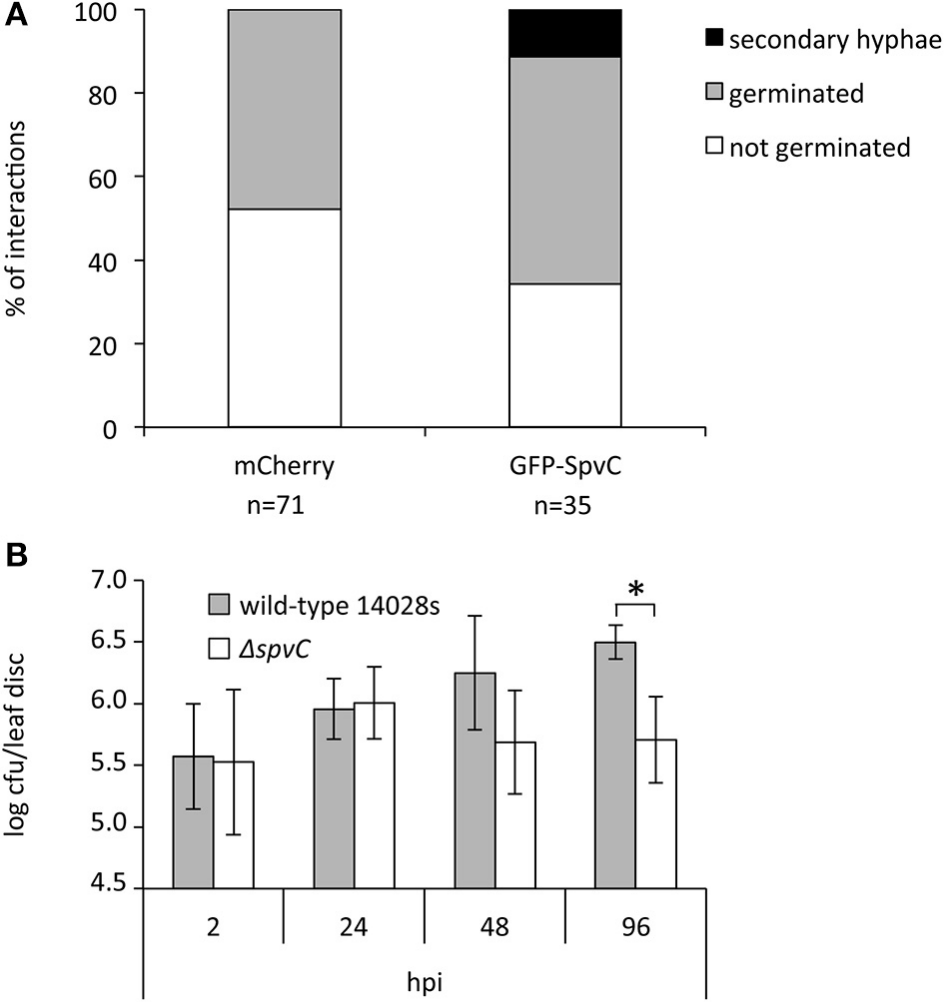
**Expression of SpvC breaches the non-host resistance in *Arabidopsis* to powdery mildew fungus and its lack renders *Salmonella* bacteria less virulent toward plants. (A)** Leaves from soil-grown *Arabidopsis* plants were co-transformed with p35S-GFP-SpvC and p35S-mCherry plasmids or transformed with p35S-mCherry plasmid alone, and inoculated with *Blumeria graminis f. sp. hordei* (*Bgh*) conidia. 48 h after inoculation, leaves were stained with calcofluor to visualize fungal growth. The outcome was counted on cells transformed with mCherry or GFP-SpvC. Three types of interaction were observed: non-germination, germination without further development, and formation of secondary hyphae. The experiment was repeated three times with similar results. **(B)** Proliferation of the Δ*spvC* mutant *in planta* was tested on 4-week old *Arabidopsis* plants, syringe-infiltrated with bacterial solutions. The data represents mean values ± SD from five biological replicates; ^*^ represents *p* < 0.05 in Student's *t*-test, hpi; hours post-infection.

MAPKs are key components of immune signaling in plants. Accordingly, we assumed that manipulation and inactivation of MAPKs by SpvC might affect plant resistance. To verify this, we analyzed the resistance of epidermal cells transformed with *SpvC* toward the fungal pathogen *Blumeria graminis f. sp. hordei* (*Bgh*). *Arabidopsis* is a non-host for *Bgh* and copes easily with this fungus either by papillae formation or by hypersensitive response (HR) at the infection site. *SpvC* was transiently expressed in *Arabidopsis* epidermal cells under the control of the constitutive 35S promoter as a GFP-tagged version (*GFP-SpvC*) and transformed leaves were inoculated with *Bgh* conidia. On control-transformed (*mCherry*) cells about 48% of *Bgh* conidia germinated 24 h after inoculation, though all of the germinated conidia died or did not develop any further in the following 24 h (Figure 6A). In contrast, in cells expressing *GFP-SpvC* the percentage of germinated conidia increased to 66% and the later developed into secondary hyphae was observed in 11% of the transformed cells (Figure 6A). These results suggest that *Bgh* successfully penetrated into part of the epidermal cells that expressed *GFP-SpvC*. We conclude that the efficient defense mechanism against *Bgh* is at least partially compromised when SpvC is present in the cell, most likely due to its effect on MAPKs and the subsequent inhibition of PTI.

### SpvC is required for full virulence of *Salmonella* toward plants

The Δ*spvC* mutant is characterized by attenuated virulence in the mouse model (Mazurkiewicz et al., 2008) and SpvC is thought to play a crucial role in systemic bacteremia in humans [reviewed in Guiney and Fierer, 2011]. To assess the question whether SpvC plays a significant role during proliferation in plants, we tested the performance of the Δ*spvC* mutant on *Arabidopsis* plants. The Δ*spvC* mutant was constructed by replacing the *SpvC* gene with a chloramphenicol resistance cassette in the wild-type *S*. Typhimurium strain 14028s (Datsenko and Wanner, 2000). Six-week old, soil-grown *Arabidopsis* plants were syringe-infiltrated and the bacterial populations were monitored during 4 days. The wild-type *S*. Typhimurium 14028s strain reached about 10^7^ colony-forming units (cfu) in a leaf disc. In contrast to the wild-type, the Δ*spvC* mutant showed a decreased ability to proliferate in *Arabidopsis* (Figure 6B), suggesting that SpvC plays a important role for *Salmonella* when present in a plant host.

## Discussion

In this report we performed a functional screen of *Salmonella* effector proteins and virulence factors in plants. We demonstrated that the function of the *Salmonella* effector protein SpvC is conserved in hosts originating from different kingdoms. In analogy to the infection in the animal system, SpvC interacts with plant MAPKs and dephosphorylates their active form, thus attenuating defense mechanisms. The presence of *SpvC* in *Arabidopsis* cells repressed the induction of several defense-related genes and breached the non-host resistance toward *B. graminis*. Moreover, the mutant lacking *SpvC* was less virulent on *Arabidopsis* plants when compared to the wild-type strain *S*. Typhimurium 14028s.

Among known *Salmonella* effectors, some are encoded on plasmids within a shared common locus called salmonella plasmid virulence (*spv*) (Boyd and Hartl, 1998). The *spv* operon is absolutely required for the development of a lethal systemic infection in the mouse model (Montenegro et al., 1991; Fierer et al., 1992; Gulig and Doyle, 1993). The expression of the *spv* operon (encoding five proteins: SpvR, A, B, C, and D) is strongly induced in intracellular bacteria and is regulated by the positive transcriptional regulator SpvR and the sigma factor RpoS (Fang et al., 1991; Krause et al., 1992). SpvC is a phosphothreonine lyase that dephosphorylates the double phosphorylated pTXpY activation loop in the kinases ERK1/2, as well as in p38 and probably JNK (Li et al., 2007; Mazurkiewicz et al., 2008; Haneda et al., 2012). In consequence, SpvC blocks the pro-inflammatory function of the MAPK pathway, facilitating the cell-to-cell spread of bacteria. In contrast to the dual-specificity phosphatases, which cleave the C-P bond, SpvC cleaves the C-O bond, promoting the formation of β-methyldehydroalanine, which cannot be re-phosphorylated. The enzymatic activity of SpvC is common to the OspF family, named after the first characterized effector protein OspF from *Shigella flexneri* (Arbibe et al., 2007; Li et al., 2007; Smith et al., 2009). Interestingly, also plant pathogens possess members of the OspF family. HopAI1 from *Pseudomonas syringe* is a close homolog to OspF/SpvC, and has similarly to the *Salmonella* protein, a phosphothreonine lyase activity. Previously, HopAI1 has been shown to dephosphorylate activated MPK3 and MPK6 in *Arabidopsis* plants (Zhang et al., 2007). Recently, MPK4 was also shown to be targeted and dephosphorylated by HopAI1 (Zhang et al., 2012).

Here, we demonstrate that SpvC dephosphorylates three activated MAPKs (MPK3, MPK4, and MPK6) in *Arabidopsis*. In both performed tests, the presence of SpvC caused loss of the phosphorylated pTEpY epitope on the MAPKs. On the one hand, the assumption that the biochemical action (cleavage of C-O bond) of SpvC on active plant MAPKs is similar to its action on pERK1/2 is very tempting, remains however to be verified. On the other hand, the dephosphorylation of MAPK3/4/6 by SpvC is clearly coupled to the attenuation of the plant defense responses. When present in *Arabidopsis* protoplasts, SpvC hinders the expression of several defense-associated genes. It also lowers the resistance of *Arabidopsis* cells against the biotrophic, non-host pathogen *Bgh*, a phenomenon observed in defense-compromised mutants [reviewed in Lipka et al., 2008)]. Similarly to the situation in animal cells, where SpvC blocks the pro-inflammatory pathway and therefore the actual defense response, inhibition of MAPKs in plants seems to block the otherwise efficient defense strategy. How specific the particular MAPKs are targeted by SpvC could not be answered. The dephosphorylation assays clearly showed the possibility to dephosphorylate MPK3, MPK4, and MPK6, a situation similar to HopAI1 (Zhang et al., 2012). Nevertheless, in contrast to MPK6, interaction of SpvC with MPK3 or MPK4 could be verified neither in BiFC nor in pull-down assays, which indicates a high affinity of SpvC toward MPK6 or that interaction with MPK3 or MPK4 requires yet other components.

During animal infection, SpvC induces late macrophage apoptosis. However, no cell death-inducing activity could be detected in plants. In contrast to macrophage apoptosis used by *Salmonella* to facilitate the cell-to-cell spread in animal organism, cellular death in plants (hypersensitive response; HR) is very often a defense mechanism induced by recognition of pathogen effector proteins by the plant intracellular R proteins. Despite the fact that SpvC is a T3SS-translocated effector in mammalian cells, the described above screen in tobacco leaves suggests that SpvC does not induce the hallmark of effector-triggered immunity (ETI) in plants, the HR, implying that SpvC is not recognized by R protein(s). We also exclude the possibility that SpvC is recognized by surface located receptors by testing its PAMP activity. Growth inhibition and production of reactive oxygen species, both hallmarks of pattern-triggered immunity (PTI), were studied in plants after contact with SpvC (Supplementary Figure S2). Our results suggest that SpvC is not toxic for plant cells when externally present and that plants do not recognize SpvC by potential surface receptor(s).

As described above, the intracellular presence of SpvC attenuated the activation of MPK3/4/6 and expression of several defense-related genes. Whether, besides inhibition of those two aspects of plant defense, SpvC actively suppresses the HR response remains to be verified in future experiments. Furthermore, the translocation of *Salmonella* effector proteins into plant cytoplasm was not yet demonstrated. The function of SpvC requires its presence in the host cytoplasm, therefore a direct evidence of translocation of this effector (or/and others) needs to be provided in future work, as this would certainly help to understand how these bacteria suppress plant immune responses. Interesting was the observation that expression of other *Salmonella* effectors *in planta* induced visible changes. SseF and SseG, both SPI-2 encoded effector proteins involved in the trafficking of *Salmonella* Containing Vacuole (SCV) in animal cells, induced HR-like (SseF) or yellowing (SseG) symptoms in tobacco leaves, when expressed via *Agrobacterium*-mediated transformation. It confirms the observation made by Ustun et al. (2012), who showed that SseF from *S. enterica* triggers HR-like symptoms in tobacco plants when expressed transiently via *Agrobacterium* infiltration or delivered via the T3SS from *Xanthomonas campestris pv. vesicatoria*. Moreover, the ability of SseF to trigger HR-like symptoms was lost upon silencing of *SGT1* (suppressor of G2 allele of skp1), which is required for HR induction in tobacco. These results indicate that *Salmonella* SseF is recognized in *N. benthamiana via* an R protein-mediated mechanism and triggers ETI in consequence. Surprisingly, expression of *SptP* or *SlrP*, both postulated to be key effectors of *Salmonella* with the highest number of predicted protein-protein interactions (Schleker et al., 2012), induced no visible symptoms in tobacco leaves nor had an effect on the induction of *pFRK1-Luc* in *Arabidopsis* protoplasts.

In summary, an increasing number of evidence indicates that plants evolved diverse mechanisms to recognize *Salmonella* bacteria using surface receptors as well as intracellular R proteins. Our study supports the view that *Salmonella* also evolved means to interfere with plant immunity by efficiently employing its repertoire of effector proteins to succumb plant immune responses. Consequently, *Salmonella*, and possibly other human pathogenic bacteria, seems to possess effective tools for suppression of the plant immune system.

### Conflict of interest statement

The authors declare that the research was conducted in the absence of any commercial or financial relationships that could be construed as a potential conflict of interest.
