# Supplementary material for: The Salmonella effector protein SpvC, a phosphothreonine lyase is functional in plant cells
Source: Front Microbiol. 2014 Oct 17;5:548. doi: 10.3389/fmicb.2014.00548 (PMC4201148; doi:10.3389/fmicb.2014.00548)
Supplement: Supplementary file 1 [file Table1.DOC]

**Supplementary Table S1. Primers used in the qPCR analysis:**

*FRK1* forward: 5’-GATGGCGGACTTCGGGTTATC-3’

*FRK1* reverse: 5’-CGAATAGTACTCGGGGTCAAGGTAA-3’

*WRKY17* forward: 5’-GCCGCTTTCTGGTCTTCCTTACAG-3’

*WRKY17* reverse: 5’-CCGTGGATGTGGTGAGCCTTTG-3’

*4CL* forward: 5’-CCCTGAGACGGAGAGATACGACTTG-3’

*4CL* reverse: 5’-TCGGTCATTCCATAACCCTGACCA-3’

*Sec61* forward: 5’-ATGCTCACTGCTTGTGCCCTATTCT-3’

*Sec61* reverse: 5’-CCGGTTCAGCTCCTTTTGTAAGTTG-3’

*actin* forward: 5’-AGT GGT CGT ACA ACC GGT ATT GT-3’

*actin* reverse: 5’-GAG GAA GAG CAT ACC CCT CGT A-3’
