# Supplementary figures and images for: The Salmonella effector protein SpvC, a phosphothreonine lyase is functional in plant cells
Source: Front Microbiol. 2014 Oct 17;5:548. doi: 10.3389/fmicb.2014.00548 (PMC4201148; doi:10.3389/fmicb.2014.00548)

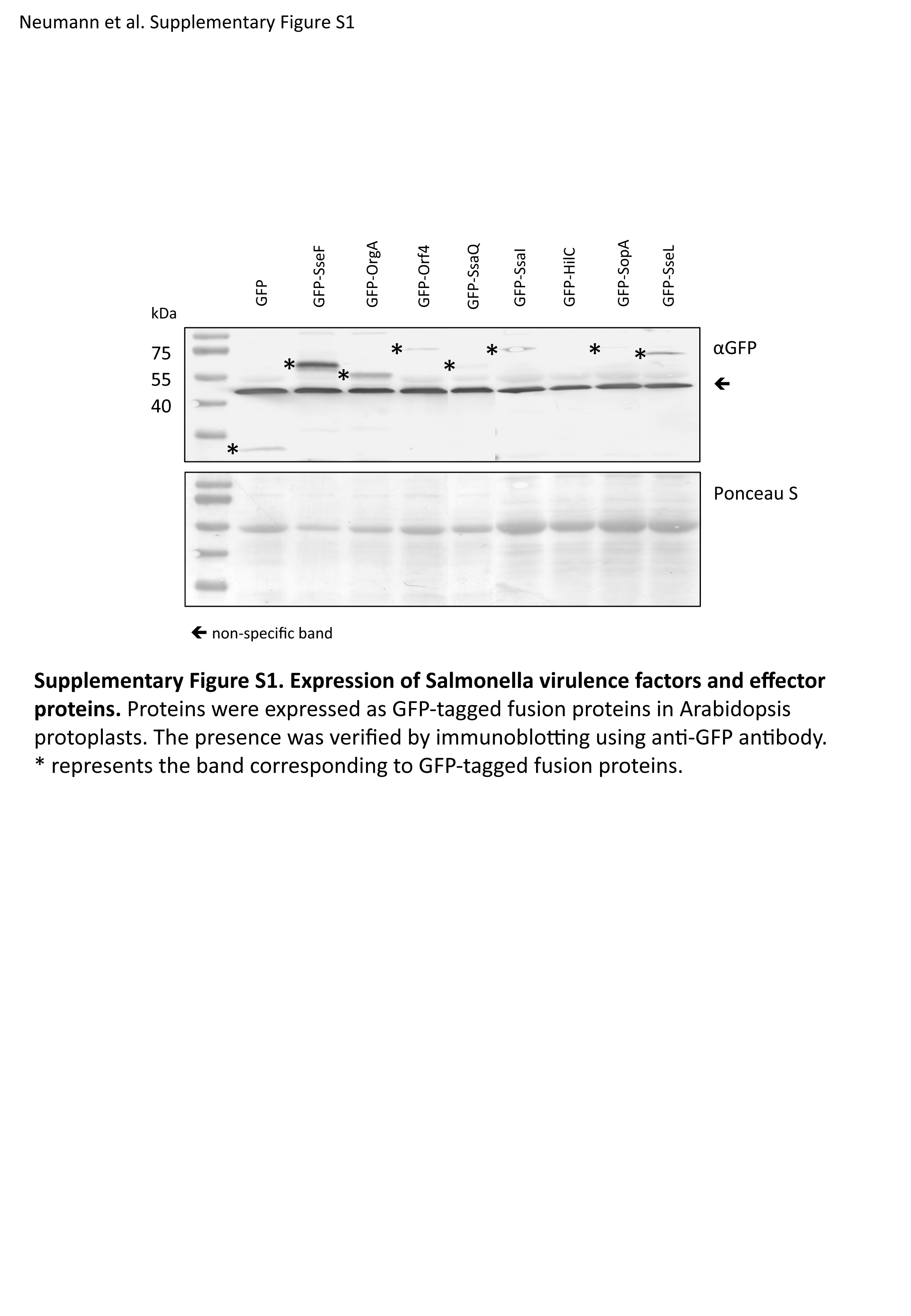

Supplement: Supplementary file 2 [file Image1.TIF]

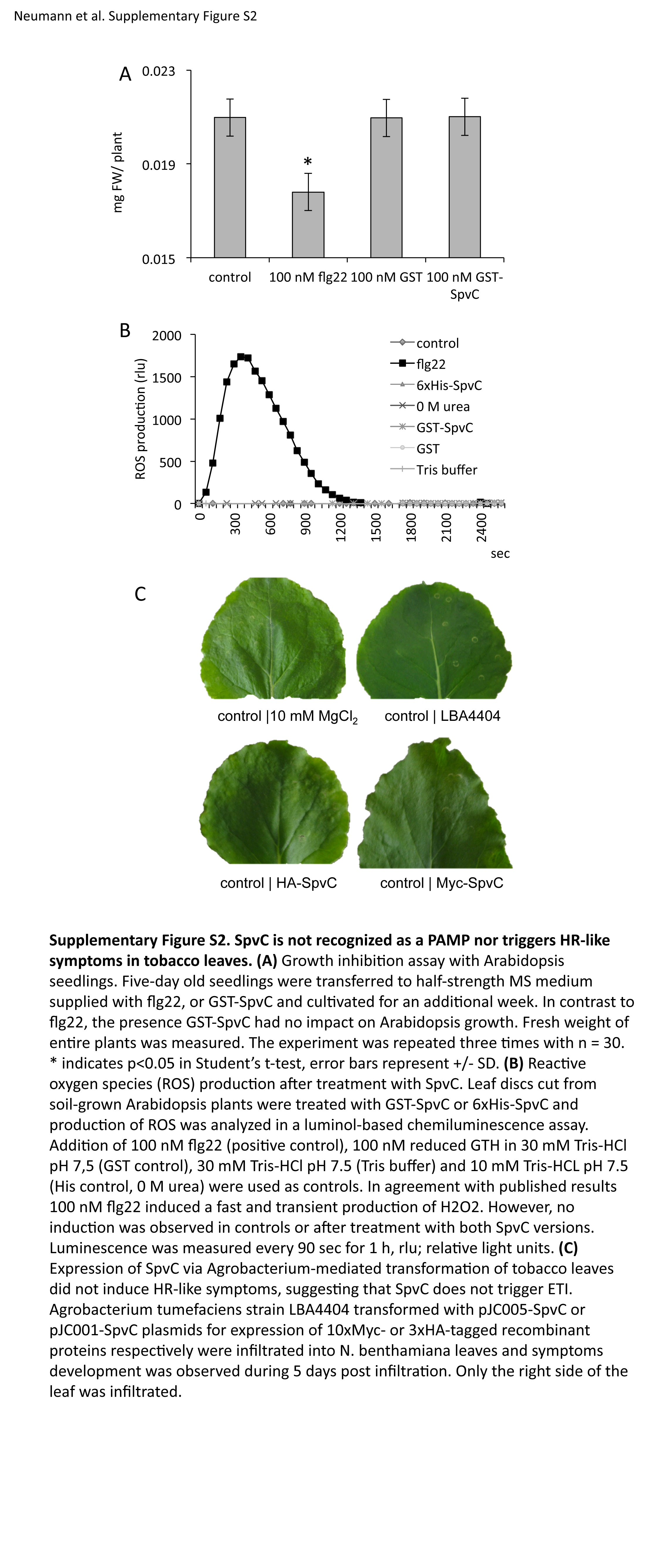

Supplement: Supplementary file 3 [file Image2.TIF]
